# Socio-cultural determinants of timely and delayed treatment of Buruli ulcer: Implications for disease control

**DOI:** 10.1186/2049-9957-1-6

**Published:** 2012-10-25

**Authors:** Mercy M Ackumey, Margaret Gyapong, Matilda Pappoe, Cynthia Kwakye Maclean, Mitchell G Weiss

**Affiliations:** 1 School of Public Health, College of Health Sciences, University of Ghana, Accra, Ghana; 2 Swiss Tropical and Public Health Institute, Basel, Switzerland; 3 University of Basel, Basel, Switzerland; 4 Dodowa Health Research Centre, Ghana Health Service, Dodowa, Ghana; 5 Amasaman Hospital, Ghana Health Service, Accra, Ghana

## Abstract

**Introduction:**

Public health programmes recommend timely medical treatment for Buruli ulcer (BU) infection to prevent pre-ulcer conditions from progressing to ulcers, to minimise surgery, disabilities and the socio-economic impact of BU. Clarifying the role of socio-cultural determinants of timely medical treatment may assist in guiding public health programmes to improve treatment outcomes. This study clarified the role of socio-cultural determinants and health system factors affecting timely medical treatment for BU in an endemic area in Ghana.

**Methods:**

A semi-structured explanatory model interview based on the explanatory model interview catalogue (EMIC) was administered to 178 BU-affected persons. Based on research evidence, respondents were classified as timely treatment (use of medical treatment 3 months from awareness of disease) and delayed treatment (medical treatment 3 months after onset of disease and failure to use medical treatment). The outcome variable, timely treatment was analysed with cultural epidemiological variables for categories of distress, perceived causes of BU, outside-help and reasons for medical treatment in logistic regression models. The median time for the onset of symptoms to treatment was computed in days. Qualitative phenomenological analysis of respondents’ narratives clarified the meaning, context and dynamic features of the relationship of explanatory variables with timely medical treatment.

**Results:**

The median time for initiating treatment was 25 days for pre-ulcers, and 204 days for ulcers. Income loss and use of herbalists showed significantly negative associations with timely treatment. Respondents’ use of herbalists was often motivated by the desire for *quick recovery* in order to continue with work and because herbalists were relatives and easily accessible. However, drinking unclean water was significantly associated with timely treatment and access to health services encouraged timely treatment (OR 8.5, p = 0.012). Findings show that health system factors of access are responsible for non-compliance to treatment regimes.

**Conclusions:**

Findings highlight the importance of an integrated approach to BU control and management considering the social and economic features that influence delayed treatment and factors that encourage timely medical treatment. This approach should consider periodic screening for early case-detection, collaboration with private practitioners and traditional healers, use of mobile services to improve access, adherence and treatment outcomes.

## Multilingual abstracts

Please see Additional file 1 for translations of the abstract into the six official working languages of the United Nations.

## Introduction

Public health programmes recommend early medical treatment for Buruli ulcer (BU) infection to prevent pre-ulcer conditions from progressing to ulcers, minimise osteomyelytis (infectious inflammation of the bone or marrow), need for surgical intervention, disability and improve treatment outcomes [1,2]. However, affected persons may delay medical treatment due to various social, economic, cultural and health system factors [3-6]. Clarifying the role of socio-cultural determinants of timely and delayed medical treatment for BU may assist in the design of public health programmes that are socio-culturally sensitive to improve disease outcomes, lessen the disease burden, and treatment costs to health facilities.

Buruli ulcer, caused by the environmental pathogen, *M. ulcerans,* is a debilitating disease of the skin and sometimes the bone tissues [5-8]. The pre-ulcer stage of infection is characterised by nodules, plaques and oedemas [8,9]. The time from progression of a pre-ulcer to an ulcer varies, ranging from a few weeks to several months [7]**.**

Until recently, wide surgical excisions that require lengthy hospital stays for recovery were the only treatment [6,10-13]. However, studies show that surgery alone cannot completely remove all necrotic tissues, and the possibility of recurrence is high [14-16]. Using antibiotics recommended by WHO – rifampin and streptomycin -for nodules and early lesions is effective in reducing lesions thus minimising the extent of surgical excision and disease sequalae [2,13,17-19].

Even though there is no standard definition for acceptable treatment delay, public health programmes encourage BU-affected persons to seek treatment as early as possible, during the pre-ulcer stage of infection, which is often characterised by a nodule, plaque, or oedema. When treatment is delayed and lesions progress into ulcers, they typically require a long time to heal, and scarring, contractures and disabilities result [6,9,11,12,20].

Studies in Benin and Ghana have investigated the obstacles to medical treatment which include, fear of recurring infections after surgical treatment, anxiety about the outcome of surgery, fear and concern about scarring and disabilities after treatment, late detection of BU-related skin trauma or lesions, perceived seriousness of infection and local beliefs of spiritual causes that require the use of traditional healers, particularly herbalists [1,4,10,19,21]. Additional obstacles to seeking medical treatment are high transport costs of seeking treatment, costs of food during hospitalisation and the social and economic implications of providing care to affected relatives during hospital admissions. [3,4,7,10,12,19,21].

It is expected that awareness and knowledge of anticipated debilitating disease outcomes of BU may prompt timely and appropriate medical treatment. However, this is not the case in many BU-endemic countries [4,12,19,21,22]. Therefore, clarifying the role of socio-cultural barriers to timely treatment for BU is likely to strengthen case-detection, improve access to treatment and outcomes, and consequently lessen disease morbidity and financial costs of surgery to health facilities. Furthermore, socio-cultural studies for BU are a priority of the WHO research agenda [2]. Nevertheless, there are too few of such studies and many were conducted before the introduction of the WHO-recommended antibiotic therapy. This paper examined socio-cultural determinants of timely medical treatment for pre-ulcers and delayed medical treatment for ulcers in an endemic area in Ghana.

## Methods

### Study area

**Figure 1:**
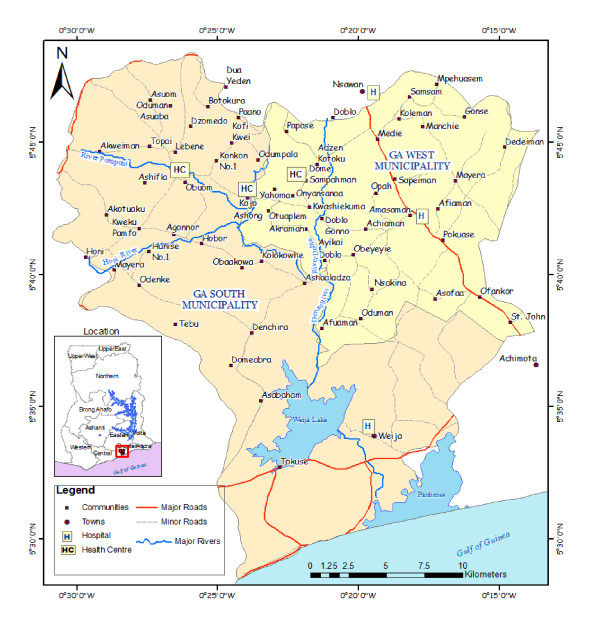
Map of the study area – the Ga-West and Ga-South Municipalities showing health facilities and study communities. *Inset is a map of Ghana showing the location of the study municipalities. To avoid overcrowding on the map, only some communities are shown.

The study was undertaken in the Ga-West and Ga-South Municipalities of the Greater Accra region from November 2008 to July 2009. The Ga-West Municipality (GWM) is predominantly rural, with a population of 215,824, based on projected population estimates from the national housing and population census (Ga-West Municipal Health Directorate, annual report, unpublished). Both municipalities cover a land area of 692 square kilometres. Seventy-six percent of the land area of the Ga-South Municipality (GSM) is predominantly urban and peri-urban while 24% is rural [23]. Health services are inaccessible to the majority of the population due to distance, terrain, poor road infrastructure and inadequate transport (Figure 1). The GWM has 1 government hospital, 5 community clinics, 9 private hospitals and clinics, and 7 private maternity homes. The major BU medical treatment centres are the Amasaman hospital (AH), which is the main referral centre for BU treatment in the Greater Accra region and the Kojo Ashong clinic. The Obom health centre provides medical treatment for BU patients in the GSM and the AH is one of the main referral centres for BU treatment in the Greater Accra region. BU ranks third on the list of top ten diseases reported at the out-patient departments in the government health facilities in both municipalities. 

### The study sample and sampling strategy

To identify as many BU affected persons as possible, a sample of 181 respondents was obtained from 67 communities and 3 BU treatment centres – The Amasaman hospital (AH), the Kojo Ashong clinic (KAC) and the Obom health centre (OHC). These health facilities provide antibiotic treatment (rifampicin and streptomycin) and surgery. The AH admits approximately 90 persons with BU infection each year. Based on these estimates, we enlisted all BU patients receiving treatment at the AH, KAC and OHC and all affected persons from 67 endemic communities. Parents acted as proxy respondents for children below 5 years of age. However, since help-seeking choices are largely determined by parents and guardians, children older than 5 years were interviewed first and subsequently parents and guardians; responses reflected consensus opinion.

### The explanatory model interview catalogue (EMIC)

A semi-structured explanatory model interview based on the EMIC framework for cultural epidemiology [24] was developed for this study. Prior ethnographic research and earlier studies [3] informed the design and the formulation of questions for the EMIC. The instruments were developed in English, but interviews were conducted in the local Ghanaian languages (Ga, Ewe and Twi) spoken by respondents in the study areas.

The EMIC examined use of timely medical treatment, patterns of distress (PD) and perceived causes (PC). Patterns of distress refer to illness-related problems and concerns and local experiences of BU illness. Perceived causes denote local ideas of causes for BU. Children were not asked PD questions that were irrelevant to their socio-cultural context. Such questions referred to marriage, income and employment. Respondents were asked to indicate the provider-type for pre-ulcers and ulcers. Those respondents who stated that they used medical treatment from recognised municipal and private health facilities were asked to indicate reasons for medical treatment and also asked to state the time lapse between onset of symptoms and medical treatment. Narratives in response to open-ended questions elaborated and explained coded categories and their responses.

### Data management and analysis

Categorical and numeric data from the EMIC interviews were double entered using EPI Info (Centers for Disease Control and Prevention, Atlanta, GA, USA, version 3.4.1), and subsequently cleaned and analysed using STATA 10.1 data analysis and statistical software (StataCorp, Lakeway Drive, College Station, Texas). Median time lag from onset of symptoms and medical treatment were recorded in the manner in which they were reported, in days, weeks, months or years and were later converted to days for analysis.

Unlike malaria and tuberculosis, there is no standard definition distinguishing delay from timely treatment for BU. The pre-ulcer phase of BU infection could vary from a few weeks to several months [25]; one study has indicated an average time of 1–3 months [9]. Host immune inflammatory response plays an important role in the progression of pre-ulcers to ulcers and therefore this was taken into consideration in the operational definition for timely treatment [26]. Timely treatment was therefore defined as seeking medical treatment for pre-ulcers and ulcers within 3 months after the onset of infection. Those respondents who initiated medical treatment 3 months after infection and those with pre-ulcers and ulcers who did not seek medical treatment were classified as delayed treatment seeking. Initially 181 respondents were interviewed but three (3) respondents who had pre-ulcers (with illness duration from 10 days to 3 weeks), were not included in the analysis because their treatment status was inconclusive. It was presumed they might seek timely or delayed treatment later.

A binary outcome variable (timely treatment) was created. To determine how features of illness explanatory models affected timely treatment, unadjusted (univariate) analysis first considered demographic, PD, PC, Help-seeking (HS) and reasons for treatment variables at p < 0.25 [27] for the outcome variable. The only exception was ‘disrupted education’ (p = 0.628) because respondents’ narratives commonly suggested the influence of this variable on treatment. Adjusted (multiple regression) models considered variables from the unadjusted analysis with p-values <0.25. Odds ratios with their respective 95% confidence intervals were calculated for variables in the model.

Narrative data were translated and transcribed in English during the interview by the data collector, entered into MS Office Word 2007 (Microsoft Corporation) and imported into MAXQDA, software for textual analysis (verbi Software Consult Sozialforschung, GmbH, Marburg, Germany). Illness narratives and quantitative variables of interest for PD, PC, HS and reasons for medical treatment were imported into MAXQDA to select respondents with a desired profile of responses for phenomenological qualitative analysis. This approach allowed clarification of the relationship of explanatory variables to timely and delayed medical treatment. The regression analysis explained which variables were related to timely or delayed medical treatment and the narratives were analysed to explain the nature of such effects**.**

### Ethical considerations

Verbal informed consent was obtained from all adult respondents and parental caretakers or guardians of children. The study was approved by the ethical review committee of the Ministry of Health, Ghana, and the ethics commission of Basel (Ethikkommission beider Basel, EKBB) in Switzerland.

## Results

### Demographic characteristics of the study respondents

Based on our operational definition of timely treatment (see methods section), 48 (27.0%) respondents initiated timely treatment and 130 (73%) respondents delayed treatment for their illness conditions. Among the 130 respondents who delayed treatment, 39 did not seek treatment for their illness conditions.

A total of 166 respondents had ulcers during the study: 37 (22.3%) initiated timely medical treatment, 91 (54.8%) delayed treatment and 38 (22.9%) did not seek treatment. Fifteen (15) respondents had pre-ulcers during the study: 11 (73.3%) used timely treatment, 1 (6.7%) delayed treatment, and 3 (20%) respondents were not included in the analysis because their treatment status was undetermined (see methods).

Most of the respondents had completed primary school (58.3% timely treatment and 51.5% delayed treatment). Respondents who were employed were mainly unskilled workers. Only 22.2% of respondents who sought timely treatment and 17.9% of respondents who delayed treatment mentioned that their income was regular (table 1).

**Table 1:** Demographic characteristics of respondents*

**Demographic characteristics**	**Timely Treatment N = 48**	**Delayed Treatment N = 130**	**P-values** **N = 178**
	**(%)**	**(%)**	
**Treatment status**			
**Sex**			0.398
Males	41.7	50.0	
Females	58.3	50.0	
**Age**			0.571
Less than 15-years-of age	56.3	44.6	
15-30 years	25.0	31.5	
30-45 years	6.3	10.8	
≥ 45 years	12.5	13.1	
**Education**			0.216
No education	14.6	26.9	
Primary	58.3	51.5	
Secondary and above	27.1	21.5	
**Occupation**			0.279
Pupil/student	64.6	52.3	
Unskilled labour	20.8	26.9	
Skilled labourer	2.1	9.2	
Unemployed	12.5	11.5	
**Income**			0.367
Regular and dependable	18.5	16.2	
Uncertain/ Cannot tell	42.7	43.1	
Irregular	38.8	40.8	
**Marital status**			0.962
Never married	70.8	67.7	
Married	22.9	23.8	
Separated, divorced and widowed	6.3	8.5	

*Results are stated in percentages. Fisher’s exact test was used for comparison between ‘timely treatment’ and ‘delayed treatment’. Timely treatment is defined as seeking treatment < 3 months from first awareness of symptoms to appropriate help-seeking.

### Illness experiences and treatment delay

The median time for initiating timely treatment was 30 days and 204 days for delayed medical treatment. table 2 shows the univariate analysis of variables for demographic characteristics and table 3 shows univariate analysis for categories of illness experience (PD), illness meaning (PC), outside-help and reasons for medical treatment variables. table 4 presents results for the adjusted (multivariate) logistic model for variables selected from the univariate analysis at p < 0.25.

**Table 2:** Unadjusted (univariate) analysis of background variables associated with timely and delayed treatment

**Socio-demographic variables**	**Timely treatment N = 178**	**P-values**
	**OR (95% CI)**	
**Sex**		
Males	Ref	
Females	1.4 (0.72, 2.73)	0.324
**Age**		
Less than 15-years-of age	Ref	
15-30 years	0.6 (0.29, 1.38)	0.249
30-45 years	0.5 (0.12, 1.74)	0.252
≥ 45 years	0.8 (0.27, 2.14)	0.601
**Education**		
Primary	Ref	
Secondary and above	1.1 (0.50, 2.45)	0.795
No education	0.5 (0.19, 1.21)	0.118
**Occupation**		
Pupil/student	Ref	
Unskilled labour	0.6 (0.28, 1.42)	0.265
Skilled labourer/Professional	0.2 (0.02, 1.47)	0.110
Unemployed	0.4 (0.08, 1.73)	0.205
Other (too young to be either employed or in school)	2.9 (0.62, 13.86)	0.176
**Income**		
Irregular	Ref	
Uncertain/ Cannot tell	1.2 (0.55, 2.52)	0.663
Irregular	1.9 (0.78, 4.67)	0.166
**Marital status**		
Never married	Ref	
Married	0.9 (0.42, 2.03)	0.833
Separated / divorced and widowed	0.7 (0.19, 2.69)	0.610

*Odds ratios, confidence intervals and p-values for all variables included in the adjusted model are shown in the table. OR = odds ratios, CI = confidence intervals Timely treatment is defined as seeking treatment < 3 months from first awareness of symptoms to appropriate help-seeking.

**Table 3:** Unadjusted (univariate) analysis of socio-cultural variables associated with timely and delayed treatment*

**Explanatory variables**	**Timely treatment N = 178**	**P-values**
	**OR (95% CI)**	
**Patterns of distress**		
Pain	0.5 ( 0.21, 1.20)	0.118
Functional disability	**0.4 (0.20, 0.91)**	**0.029**
Disrupted education	0.8 (0.44, 1.65)	0.628
Loss of income	**0.4 (0.20, 0.92)**	**0.030**
Anxiety	0.6 (0.33, 1.27)	0.204
Embarrassed about condition	0.6 (0.29, 1.11)	0.098
Recurring infection	3.0 (0.90, 9.65)	0.073
**Perceived causes**		
Drinking unclean water	1.6 (0.83, 3.21)	0.158
Prone to illness	0.5 (0.16, 1.54)	0.229
Weakness of blood	0.6 (0.30, 1.26)	0.186
**Outside-help**		
Herbalist	**0.3 (0.15, 0.60)**	**0.001**
Fetish/spiritualist	**0.2 (0.07, 0.84)**	**0.025**
Prayer camp	0.4 (0.16, 1.08)	0.071
Municipal health centres	**2.7 (1.24, 5.88)**	**0.012**
Government hospital outside the district	0.6 (0.22, 1.49)	0.252
Nothing	0.3 (0.06, 1.13)	0.073
**Reasons for medical treatment**		
Easy access to health centre	**8.3 (2.46, 27.94)**	**0.001**
Self-referral	**2.3 (1.13, 4.57)**	**0.022**
Referral by family and friends	**2.9 (1.39, 6.09)**	**0.005**
Get well quickly	**3.7 (1.52, 8.79)**	**0.004**
Effectiveness of antibiotic treatment	**3.1 (1.38, 6.88)**	**0.006**

*Only variables with p-value <0.25 are shown in the table except disrupted education as a pattern of distress, because it was often mentioned in respondents’ narratives with reference to medical treatment. OR = odds ratios, CI = confidence intervals and p-values for all variables included in the adjusted model are shown in the table. Values in bold indicate statistical significance (p ≤ 0.05). Timely treatment is defined as seeking treatment < 3 months from first awareness of symptoms to appropriate help-seeking.

**Table 4:** Adjusted (Multivariate) analysis of background variables and socio-cultural variables associated with timely and delayed treatment *

**Treatment status**	**Timely treatment N = 178**	**P-values**
	**OR (95% CI)**	
**Education**		
Primary	Ref	
Secondary and above	0.5 (0.16, 1.62)	0.254
No education	0.4 (0.09, 1.58)	0.180
**Patterns of distress**		
Problems with mobility and use of affected limbs	0.8 (0.29, 2.50)	0.762
Disrupted education	0.4 (0.12, 1.63)	0.220
Loss of income	0.5 (0.13, 1.75)	0.267
Anxiety	0.6 (0.25, 1.57)	0.316
Embarrassed about condition	0.6 (0.22, 1.41)	0.216
Recurring infection	3.5 (0.71, 17.63)	0.125
**Perceived causes**		
Drinking unclean water	**3.8 (1.34, 10.63)**	**0.011**
Prone to illness	0.2 (0.05, 1.09)	0.064
Weakness of blood	0.6 (0.24, 1.79)	0.406
**Outside-help**		
Herbalist	**0.2 (0.08, 0.56)**	**0.002**
Fetish/spiritualist	0.2 (0.05, 1.09)	0.064
Prayer camp	0.4 (0.13, 1.32)	0.136
Municipal health facilities	1.2 (0.31, 4.68)	0.792
Government hospital outside the district	0.3 (0.05, 1.56)	0.150
Nothing	0.4 (0.04, 2.96)	0.343
**Reasons for medical treatment**		
Easy access to health centre	**8.5 (1.61, 44.47)**	**0.012**
Self-referral	2.3 (0.74, 6.98)	0.151
Referral by family and friends	1.6 (0.57, 4.43)	0.374
Get well quickly	1.5 (0.30, 7.32)	0.620
Effectiveness of antibiotic treatment	2.8 (0.55, 14.51)	0.215

*Odds ratios, confidence intervals and p-values for all variables included in the adjusted model are shown in the table. Values in bold indicate statistical significance (p ≤ 0.05). The fitness of the model was assessed with the p-value (p < 0.001). OR = odds ratios, CI = confidence intervals. Timely treatment is defined as seeking treatment < 3 months from first awareness of symptoms to appropriate help-seeking.

None of the demographic variables showed any significant association with timely treatment (table 2). However, functional disability and income loss as illness experiences were negatively associated with timely treatment only in the univariate analysis (table 3).

In their narratives, respondents linked income loss with the inability to continue working while seeking treatment. Respondents who delayed medical treatment also explained how their desire to continue with work overshadowed the need to use medical treatment. Many used herbalists and purchased analgesics, blood tonics and antibiotics from local chemists. They mentioned that the key reason for considering medical treatment, after herbal treatment had failed, was to get better to continue working. Most parents and guardians were unskilled workers and earned meagre wages. They expressed a genuine difficulty in stopping work to take their children and wards for medical treatment. However, respondents with pre-ulcers who initiated timely treatment continued working since they did not experience pain or much discomfort.

### Perceived causes and treatment delay

From the univariate analysis, PC variables did not show any significant relationship with timely treatment. However after adjusting for confounding factors (such as education, PD variables, help providers aside from herbalists, reasons for medical treatment besides easy access to treatment), drinking unclean water as a PC, was significantly associated with timely treatment (OR 3.8, p = 0.011) in the multivariate analysis (table 4). Respondents who linked their illness to drinking unclean water attributed this knowledge to messages from health staff. They bemoaned the absence of potable water in their communities and explained that they often fetched water from rivers, ponds and unprotected dams, (which animals also drank from), for domestic use. Sometimes rivers were used as thoroughfare to work and school. The following narrative explains the use of unclean water from a stream for drinking and as an access route to school:

I believe it is due to wading, fishing and drinking water from the Doblo stream which I drink often. I also go fishing in the stream. Sometimes I have to wade through the same stream on my way to school and the farm.

(15-year-old male respondent)

### Help-seeking behaviour

**Figure 2:**
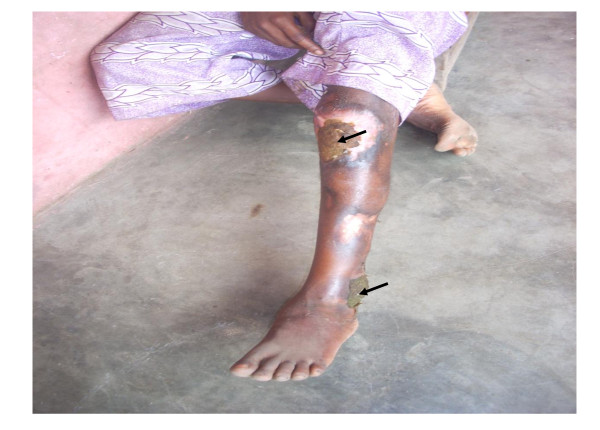
Herbal treatment for ulcers*. *Respondent has had BU for more than 3 years and is being treated at home by his grandfather, a herbalist. Respondent’s current condition is from recurring BU infections. The green patches (arrowed) are herbal dressings. Note the multiple scarring. Picture taken by Mercy Ackumey in the Otuapleam community, 2008.

Prior use of a traditional healer (herbalist and spiritualist) showed a negative association with timely treatment in the unadjusted model (table 3). Furthermore, the use of herbalists showed significantly negative associations with timely treatment after adjusting for confounding (OR, 0.2, p = 0.002), (table 4). Respondents’ use of herbalists was often motivated by the desire for *quick recovery* in order to continue with work. Narratives suggested that herbalists were relatives (Fathers, Uncles or Grandfathers), itinerant, and they lived nearby, thus making them easily accessible (Figure 2). During interviews, there were occasional encounters with herbalists. They either came to review the BU-illness status of relatives or clients, or they were carrying out their itinerant business.

Respondents had initial confidence in the claims of herbalists about their ability to treat BU. They often explained that herbalists were good at exposing the *cotton wool,* (translation from local name for infected tissues) but were not very effective in treating the sores. Respondents, who delayed treatment for ulcers, indicated that herbal treatment was often used in combination with analgesics, antibiotics and balms obtained from drug peddlers or used alone.

Respondents resorted to medical treatment, when herbal treatment seemed *ineffective* and wounds were not healing fast enough. The following narrative explains how the dynamics of easy access to herbal treatment and assurances from the herbalist, coupled with the desire to resume work, influenced the respondent’s choice for herbal treatment which subsequently delayed medical treatment. This respondent lives 8 kilometres away from the nearest health centre.

*I applied herbs to the boil at home and later invited a herbalist to treat me at home, because my treatment was not working. The herbalist assured me that his treatment was effective. His treatment only removed the ‘cotton wool’,* (translation from the local name for infected tissues) *but left a very big sore. After herbal treatment a private practitioner was injecting me and giving me pills daily at home. I always felt dizzy after the injections. I wanted to be treated at home to recover quickly to go back to work. I have spent so much money treating this disease and I have not been able to work for 9 months since I had this condition. I am a hairdresser and I have lost all my customers.*

(35-year-old female respondent)

### Access to health facilities, knowledge of antibiotic treatment influence of family and friends and medical treatment

Self-referral, referral by family and friends, the desire for quick recovery and knowledge of the effectiveness of antibiotic treatment showed significant associations with timely medical treatment in the univariate analysis (table 2). However, after adjusting for confounding only easy access to health facilities showed a significant association with timely medical treatment (OR 8.5, p = 0.012), (table 4). Respondents who accessed health centres with very little difficulty lived nearby and commuted easily for treatment. Respondents attributed their knowledge of the availability and effectiveness of antibiotic treatment at medical facilities to community health education programmes. They also conferred with family and friends for advice on treatment choices. In some cases, family and friends advised the use of medical treatment; others advised otherwise. The following narrative shows how advice from family could influence behaviour and possibly lead to delayed treatment, with implications for emotional, physical and financial distress.

*This condition has caused me a lot of inconvenience. It* (sore) *smells so bad and I have lost the desire for food. I cannot sit on my bottom* (locus of the sore) *for months. I cannot explain my situation; I am in a total mess. I don’t work anymore so I don’t have any income. I have left the family behind at home and I am in the hospital. Men cannot take care of children properly so I worry about the situation in the house. I was advised by so many people – family and friends. Any time someone advises me to try something I do it. I tried all kinds of herbs, pills and balms. I also went for prayers. My Pastor said I should go to the hospital so that my condition does not become worse.*

(32-year-old female respondent)

### Challenges associated with treatment adherence

Aside from the desire to continue with work, narrative accounts of respondents identified the influence of other socio-economic factors responsible for treatment delay. These included the cost of food if admitted to the hospital and transport expenses to medical facilities. Many of these respondents, who delayed medical treatment, described these costs as enormous, which their meagre incomes from small-scale farming, other farm work, odd-jobs and petty trading could not support. They stated that they had to leave behind some money for the family upkeep when admitted for surgery at the hospital.

Of the 91 respondents who delayed medical treatment for ulcers, nine (9.9%) could not adhere to treatment. Reasons given were distance to the health centre, high costs of transport, difficulty in obtaining transport, dissatisfaction with slow-healing of antibiotics, lack of money for food while on admission, advice of family to discontinue treatment and the perceived ineffectiveness of medical treatment influenced by the idea that BU is caused by witchcraft.

Nine (9) of the 48 respondents (18.5%) who initiated timely medical treatment for their pre-ulcer conditions failed to adhere to treatment regimes. Some of them discontinued treatment and resorted to self medication with antibiotic capsules, particularly Terramycin and Phenoxymethylpenicillin (commonly known as penicillin v), which were purchased from chemist shops or itinerant drug peddlers. Explanations were based on difficulty obtaining transport to health centres, long distance to health centres from place of residence, travel time interfering with work schedules and lack of money for transport. The following account of a respondent, an itinerant petty trader, who lives 18 kilometres from the nearest health centre, is characteristic:

I wanted to get well quickly as the health people have been telling us. One day when I was selling, I met some people from the hospital giving a talk about Buruli ulcer. When I showed them my boil they said it was Buruli ulcer and they asked me to go to the Kojo Ashong clinic for treatment. Everyday, I had to walk for a long time to get to the Kojo Ashong clinic. I was given injections and pills. I did not have enough time to take care of the family before leaving home. The clinic is far away from my house and so it was very difficult to go each time. I come back from the clinic very tired, and then I have to go and sell.

(28 year-old female respondent)

## Discussion

The aim of this study was to clarify the influence of socio-cultural factors on timely treatment for BU infection. Because of the absence of a standard definition for measuring timely treatment for BU, we formulated a working definition of timely treatment as medical treatment within 3 months of awareness of infection. This definition was based on studies that estimated an average time of 1–3 months for the pre-ulcer phase of BU [1,25]. Our findings suggest that timely treatment for BU is greatly influenced by health system factors, poverty and the socio-cultural environment of affected persons. Access to health services, referral by family and friends and awareness of the effectiveness of medical care encouraged timely treatment. Furthermore, our findings confirm earlier studies that explained delayed medical treatment for BU as a result of social and economic factors, such as the absence of reliable transport to health facilities, high costs of transport to medical facilities, prolonged stay in the hospital and loss of income, and disrupted education [3-5,10,12,21,22,28].

Previous studies have established an association between witchcraft as a PC and delayed medical treatment after prior use of traditional healers, particularly diviners. [4,21,22]. However, our findings did not show any indication of such an association between local ideas of witchcraft delaying timely treatment. Nonetheless, the use of spiritualists and herbalists for treatment had a negative association with timely treatment. Spiritualists and herbalists were frequently used however because they were easily accessible and provided home-based care which minimised absenteeism from home for medical treatment.

### Study limitations, strengths and implications for control

Recall bias may have been an issue since study data were mainly based on respondents’ accounts. Because many respondents had to report both on providers visited and time when they initiated treatment retrospectively, recall bias is a potential problem. Probes were used however, to minimise recall bias and under-reporting. We interviewed fewer respondents with pre-ulcers (15) compared with those who had ulcers (166). The skewed nature of our data can be explained by local practices of incising nodules and applying herbs to pre-ulcer lesions which accelerates the progression of pre-ulcer lesions to ulcers. A recent study in a BU-endemic area in Ghana also showed fewer pre-ulcer cases (23.3%) than ulcer cases (76.7) during an initial health-screening exercise. However, the situation reversed after one year of intensive health education[29]. The impact of this practice has been reported in another paper [30].

Narratives explaining the influence of cultural epidemiological explanatory variables clarified the socio-cultural context of timely treatment and enhanced the social contextual analysis of logistic regression models. Findings provide insight into features of both timely and delayed treatment for BU and indicate programme-relevant issues for control. These include improving access to medical treatment and fostering provider-patient interactions through mobile services, involving private health care practitioners to improve access and strengthening support networks to raise awareness and provide emotional support. These points are discussed in greater detail in the discussion that follows.

### Improved access to services for timely treatment and adherence

A study in Benin reported a shorter median time delay of 120 days for ulcers compared with 204 days in our study [7]. The median time for delayed treatment and the long duration of infection for ulcers is a matter of concern because this might lead to prolonged treatment with higher costs and disability that deepens poverty [3,5,7,28]. Based on the median time for initiating timely treatment in our study, which was 30 days and the average time for incubation for *M. ulcerans* (between 1–3 months) [9], we suggest that persons infected with *M. ulcerans* infection, should seek medical treatment within a month after awareness of symptoms. It must be noted however that improved access to treatment is required to encourage affected persons to seek treatment within a month of infection,

In Benin, median time delay for BU decreased from 120 days to 30 days after the implementation of a programme to improve access to care [7]. Study findings indicated that although proximity of health facilities to residences encouraged timely care, distance, travel time to health facilities that disrupted work schedules, lack of money for transport, unavailability of transport and loss of wages when seeking care accounted for non-adherence to a full course of antibiotic treatment. A study in Ghana revealed that low income also accounted for non-adherence to tuberculosis treatment. People defaulted when they felt a bit better in order to work and continue taking care of the family [31]. Transport costs and distance from health facilities have been responsible for treatment delays and adherence for tuberculosis [32]. Our study finding which confirms this link between access, poverty and disrupted livelihoods, on the one hand, and non-adherence to antimicrobials is a matter of concern; it is likely to increase antibiotic resistance and compromise effective treatment [33].

It is important that public health practitioners consider the socio-economic conditions of BU-affected persons. These conditions have implications for designing programmes and providing services to improve disease outcomes, lessen disease burden, limit dependency on herbalists and encourage timely treatment, and mitigate the effects of poverty. Mobile services are likely to increase interactions between BU patients and health workers, which are crucial in motivating commitment to treatment, providing emotional support and encouraging adherence to antibiotic treatment regimes for positive treatment outcomes, namely cure and reduced recurrences.

The use of motorcycles to improve access to health services is not a new phenomenon in Africa. Motorcycle ambulances have been used in Malawi to improve access to health facilities, improve referrals and consequently reduce maternal mortality [34]. In South Africa, off-road motorcycles have been used for timely collection of blood-specimens that give remote clinics access to diagnostic laboratory services [35]. Collaborating with private health practitioners might also be considered as a pragmatic and cost-effective approach to improve access [30,36]. However, this intervention requires supervision and monitoring by the municipal health management team to ensure that drug protocols are followed strictly and wounds are managed properly.

### The socio-cultural context of poverty and timely treatment

The failure to initiate timely medical treatment was associated with concern for securing livelihoods. Most BU-affected persons are poor, unskilled labourers, petty traders, farmers or fishermen, with irregular work schedules and incomes [3,12,28,29,37]. Their concern about loss of livelihoods and income is reasonable and well-founded. Studies have shown the immense socio-economic burden of BU on already impoverished families and households [12,19,37]. Some families, borrow money, sell assets and reduce farm sizes to pay for transportation and feeding costs related to BU treatment, thus entrenching them deeper into poverty [28,29]. Ironically, however, treatment delays account for longer periods of treatment, higher cost, longer hospitalisation, loss of livelihoods and increased poverty [29].

### Influence of use of herbalists on timely treatment

Many respondents who delayed treatment for ulcers had first used herbalists (43.1%). Herbal treatments and herbalists are used widely for various ailments in Ghana [38]. The use of herbalists however, is known to delay medical treatment for BU [1,3] and tuberculosis [39]. The pre-ulcer stages of BU infection are usually without pain and unless secondary infection is introduced, ulcers are generally painless [20], which makes it easier to delay treatment to continue working.

The consanguine relationship of herbalists with our study respondents and the itinerant nature of their services made them easily accessible. Herbalists play a crucial role in providing services when biomedical treatment is inaccessible, particularly in a culture where herbal medicine is widely used [38]. Previous studies recommend their integration in the health system to facilitate referrals [3,30]. Currently, herbalists are being motivated to refer patients to health facilities, in the study areas (personal communication). This strategy, although laudable needs to be explored further to consider innovative ways to enlist their trust and cooperation.

### Perceived causes and timely treatment

Respondents who initiated timely medical treatment were more likely to attribute the cause of their illness to drinking bad water. Even though the mode of BU transmission to humans is unclear [40], support is weak for the assertion that ingestion of unclean water is a possible transmission route [41]. Local perceptions that link BU disease to drinking unclean water may result from misinterpretation of health messages that emphasise water contagion as a risk factor for BU disease. Furthermore, the study location was a previously guinea worm endemic area and health education messages then emphasised drinking unclean water as a risk factor. Therefore, there is a tendency of generalising health messages across these two diseases. Public health programmes must consider the disease history of communities when designing health education programmes and present messages distinctly to avoid ambiguity. Nonetheless, study findings support the concern raised by earlier studies about the need for further research on the role of environmental factors, animals and insects in BU contagion [25,42,43]. Such evidence would maybe help to guide and motivate the local population to clarify the validity of problems that affect timely medical treatment.

### Support networks and health education for timely treatment

Self-referral, referral by family and friends and knowledge of WHO-antibiotic treatment was significantly associated with timely treatment only in the univariate analysis (table 3). Support networks such as family and friends provide social support and offer valuable help-seeking advice. [4,30,44,45]. The importance of community and school health education programmes to increase awareness of BU, and the availability and effectiveness of treatment at medical facilities cannot be overemphasised.

Health education should also explain how initial use of herbal treatment and self-medication delays medical treatment and healing. Furthermore, health messages should emphasise the effectiveness of WHO-recommended antibiotics for treating timely lesions.

## Conclusions

Our findings highlight how health system factors such as access to treatment and knowledge about the effectiveness of medical services encouraged timely treatment, and how poor access to treatment and socioeconomic obstacles affected treatment adherence. The socio-cultural context of poverty discouraged timely treatment and influenced use of herbalists. The link between drinking water as a perceived cause and timely treatment is a clear indication of a positive response to health messages and shows the impact of the control programmes even through the scientific validity of this perceived cause is questionable. Nevertheless, findings highlight the importance of optimising public health control efforts. This calls for an integrated approach to BU management and care accounting for social and economic barriers to timely medical treatment. Recommended measures include, periodic screening for timely case-detection, collaboration with private practitioners to improve access to treatment and the introduction of mobile services to improve treatment outcomes and adherence, and case-detection.

## Competing interests

The authors declare that they have no competing interests.

## Authors' contributions

MMA designed the study, collected and analysed field data and wrote the manuscript. MG and MP assisted in study design and editing of the manuscript. CKW assisted in the editing of the manuscript. MGW also designed the study, assisted with data analysis and editing of the manuscript. All authors read and approved the final manuscript.

## Supplementary Material

Additional file 1 Multilingual abstracts in the six official working languages of the United Nations. Click here for file
