# Supplementary material for: Socio-cultural determinants of timely and delayed treatment of Buruli ulcer: Implications for disease control
Source: Infect Dis Poverty. 2012 Oct 25;1:6. doi: 10.1186/2049-9957-1-6 (PMC3710079; doi:10.1186/2049-9957-1-6)
Supplement: Additional file 1 — Multilingual abstracts in the six official working languages of the United Nations. [file 2049-9957-1-6-S1.pdf]

Translation of the abstract into the six official working languages of the United Nations

الآثار المترتبة على مكافحة الأمراض :المحددات الاجتماعية و الثقافية لعلاج قرحة بورولي في الوقت المناسب و المتأخر

ماكلين وميتشل جي فايس-نثيا كواكيميرسي إم أكوميي، مارغريت جيابونج،ماتيلدا بابو ، سيد

ملخص

للحيلولة من تطور حالات (BU) برامج الصحة العامة توصي بالعلاج الطبي في الوقت المناسب للإصابة بقرحة بورولي :مقدمة
ر المحددات توضيح دو .الاقتصادي لقرحة بورولي-مقدمات القرحة أن تصبح قُرح، للتقليل من الجراحة، والإعاقة والأثر الاجتماعي
و .الثقافية للعلاج الطبي في الوقت المناسب يمكن أن تساعد في توجيه برامج الصحة العامة من أجل تحسين نتائج العلاج-الاجتماعية
الثقافية و عوامل النظام الصحي التي تؤثر على العلاج الطبي في الوقت المناسب -أوضحت هذه الدراسة دور المحددات الاجتماعية
.رولي في منطقة موبوءة في غاناالقرحة بو

شخصاً 178 قُدِم إلى (EMIC) نموذج مقابلة تفسيرية شبه منظمة استناداً إلى قائمة نموذج المقابلات التفسيرية المصورة :الأساليب
باستعمال العلاج الطبي) استناداً إلى الأدلة البحثية، صُنِف المستجيبون بأنهم تعاطوا علاج في الوقت المناسب .مصابين بقرحة بورولي
.(أشهر بعد الإصابة بالمرض وعدم استخدام العلاج الطبي 3 العلاج الطبي) و آخرون تأخر علاجهم (أشهر من اكتشاف المرض 3 بعد
لقرحة النتائج متغيرة، حُلِلَ العلاج في الوقت المناسب باستخدام المتغيرات الوبائية الثقافية لفئات شدة المرض، الأسباب المتصورة
تم حساب متوسط وقت ظهور الأعراض للعلاج .بورولي، المساعدة الخارجية و أسباب العلاج الطبي في نماذج الانحدار اللوجستية
تحليل الظواهر النوعية من روايات المستجيبين وضحت السياق المعنيّ و الخصائص الديناميكية للعلاقة بين المتغيرات .خلال أيام
.ج الطبي في الوقت المناسبالتفسيرية مع العلا

وأظهر فقدان الدخل واستخدام الأعشاب .للقرح 204 يوماً بالنسبة لمقدمات القرحة، وأيام 25 وكان متوسط الوقت لبدء العلاج :النتائج
ي تحقيق الشفاء السريع غالباً ما كان استخدام المستجيبين للأعشاب بدافع الرغبة ف .متلازمات سلبية كبيرة مع العلاج في الوقت المناسب
ومع ذلك، شرب مياه غير نظيفة ارتبط إلى حد .من أجل الاستمرار في العمل، ونظراً لأن الأعشاب قريبة ويمكن الوصول إليها بسهولة
و تبين .(8.5OR, p=0.012) كبير بالعلاج في الوقت المناسب والوصول إلى الخدمات الصحية شجع العلاج في الوقت المناسب
.تائج أن عوامل الوصول للنظام الصحي هي المسؤولة عن عدم الامتثال لنظم العلاجالن

وتبرز النتائج أهمية اتباع نهج متكامل لمراقبة قرحة بورولي و التعامل معها مع الأخذ في الاعتبار السمات الاجتماعية و :الاستنتاجات
هذا النهج ينبغي أن يهتم بالفحص .جع على العلاج الطبي في الوقت المناسبالاقتصادية التي تؤثر في تأخر العلاج و العوامل التي تشد
الدوري للكشف المبكر عن الحالة، والتعاون مع الممارسين في القطاع الخاص والمعالجين التقليديين، واستخدام خدمات الهاتف النقال
.لتحسين الوصول للعلاج والالتزام به ونتائج العلاج

Translated from English version into Arabic by Berlant Tosson, through

TRANSLATORS
WITHOUT BORDERS

影响布鲁里溃疡患者就医时间的社会文化因素及其对疾病控制的影响

Mercy M Ackumey, Margaret Gyapong, Matilda Pappoe, Cynthia Kwakye-Maclean and Mitchell G Weiss

摘要

引言：公共卫生计划建议布鲁里溃疡患者应尽早就医，防止病情由溃疡前期恶化至溃疡，以减少外科手术次数、以及该病引起的残疾和对社会经济的影响。阐述社会文化因素对患者及时就医的影响可能有助于指导公共卫生计划，改善治疗效果。本研究阐述了加纳某流行区社会文化因素和卫生体系相关因素对布鲁里溃疡患者及时就医的影响。

方法：基于解释性面试模型目录（EMIC），建立半结构化解释性面试模型，对 178 名布鲁里溃疡患者进行调查。通过调查，将被调查者分为及时就医（发病 3 个月内就医者）和延迟就医（发病 3 个月后就医者和未就医者）。采用 Logistic 回归模型，以及时就医为结果变量，分析其与文化方面和流行病学方面的变量，如疾病分型、感知的病因、外界的帮助和就医的原因等的相关性。自发病到就医的时间中位数以天数计算。根据被调查者的叙述，现象学上的定性分析阐明了及时就医与解释变量间的关系是有意义的，且具有动态的特征。

结果：溃疡前期的患者就医时间的中位数为 25 天，溃疡患者就医时间的中位数为 204 天。收入减少和使用草药与及时就医呈显著负相关。被调查者使用草药通常是出于想快速恢复，能持续工作，以及因为草药医生是亲戚，方便就诊。然而，饮用不干净的水与及时就医显著相关。就医的方便程度也与及时就医显著相关(OR 8.5, p=0.012)。研究结果显示，患者对治疗方案的依从性与就医的方便程度有关。

结论：鉴于社会经济因素对治疗延误的影响，以及促进及时就医的相关因素，对布鲁里溃疡防治和管理需要一个综合性的方法。该方法需包括对早期病例的周期性筛查，与私立医药从业者和传统治疗者的合作，以及移动服务的使用，以改进就医的方便程度，治疗方案的执行和效果。

Translated from English version into Chinese by Qu Lin-Ping, through

Les déterminants socioculturels du traitement précoce et tardif de l'ulcère de Buruli : leurs implications dans la lutte contre la maladie

Mercy M Ackumey, Margaret Gyapong, Matilda Pappoe, Cynthia Kwakye-Maclean et Mitchell G Weiss

Résumé

Introduction : Les programmes de santé publique recommandent un traitement médical précoce de l'ulcère de Buruli (UB), ceci afin d'éviter l'évolution de la phase pré-ulcéreuse en ulcère, de réduire au maximum la nécessité de recourir à la chirurgie et de limiter les incapacités dues à l'UB ainsi que son impact socioéconomique. Clarifier le rôle des déterminants socioculturels intervenant dans la précocité du traitement médical pourrait permettre d'orienter les programmes de santé publique en vue d'améliorer les résultats des soins. Cette étude clarifie le rôle des déterminants socioculturels et des facteurs du système de santé affectant la précocité du traitement de l'UB dans une zone endémique au Ghana.

Méthodes : 178 personnes affectées par l'UB ont été évaluées dans le cadre d'un entretien semi-structuré explorant les modèles explicatifs, fondé sur l'Explanatory model interview catalogue (EMIC). En se basant sur les résultats de la recherche, les personnes interrogées ont été classées en fonction du traitement : précoce (recours au traitement médical sous 3 mois après avoir pris connaissance de la maladie) et tardif (traitement médical 3 mois après l'apparition de la maladie et non-recours aux soins médicaux). Variable dépendante, le traitement précoce a été analysé en fonction de variables épidémiologiques culturelles pour les catégories de détresse, causes perçues de l'UB, aide extérieure et motifs du traitement médical dans les modèles de régression logistique. La durée moyenne de l'apparition des symptômes au traitement a été calculée en jours. L'analyse qualitative phénoménologique du récit des personnes interrogées a clarifié le contexte de signification et les éléments dynamiques de la relation entre les variables explicatives et le traitement médical précoce.

Résultats : la durée moyenne pour initier le traitement a été de 25 jours pour la phase pré-ulcéreuse et de 204 jours pour les ulcères. Des liens négatifs clairs ont été mis en évidence entre la perte de revenus, le recours aux herboristes et le traitement précoce. Le recours des personnes interrogées aux herboristes a souvent été motivé par le désir d'un prompt rétablissement en vue de continuer à travailler, et par le fait que les herboristes étaient des proches et étaient facilement accessibles. Cependant, la consommation d'eau

insalubre a été significativement associée au traitement précoce, et l'accès aux services de santé a contribué à la précocité du traitement (OR 8,5, p=0,012). Les résultats montrent que les facteurs d'accès au système de santé sont responsables de la non-observance des traitements.

Conclusions : Les résultats ont mis en évidence l'importance d'une approche intégrée du contrôle et de la gestion de l'UB prenant en considération les caractéristiques sociales et économiques influençant le traitement tardif, ainsi que les facteurs favorisant un traitement médical précoce. Cette approche devrait envisager un dépistage périodique pour une détection précoce des cas, la collaboration avec des praticiens privés et des tradipraticiens, le recours à des services mobiles pour améliorer l'accès, l'observance et les résultats du traitement.

Translated from English version into French by Amandine D., through

TRANSLATORS
WITHOUT BORDERS

Социокультурные детерминанты своевременной и несвоевременной терапии язвы Бурули: последствия для контроля заболевания

Mercy M Ackumey, Margaret Gyapong, Matilda Pappoe, Cynthia Kwakye-Maclean and Mitchell G Weiss

Автореферат

Введение

В рамках программ обеспечения общественного здравоохранения рекомендуется своевременное лечение инфекций, приводящих к язве Бурули (ЯБ), для предотвращения трансформации предъязвенных состояний в язвы, уменьшения масштабов хирургического вмешательства и инвалидизации, а также социоэкономического эффекта ЯБ. Прояснение социокультурных детерминантов своевременного медицинского лечения может помочь в ориентации программ обеспечения общественного здравоохранения на улучшение результатов лечения. Данное исследование проясняет социокультурные детерминанты и внутренние факторы в системе здравоохранения, влияющие на своевременность лечения ЯБ в эндемичной области республики Гана.

Методы

Проинтервьюированы 178 респондентов, пораженных язвой Бурули, по методу полуструктурированного интервью с объяснительной моделью, основанного на каталоге объяснительных моделей. На базе полученных в ходе исследования результатов респондентам были присвоены классификации «получивших своевременное лечение» (получивших медицинскую помощь в течение 3 месяцев с момента диагностирования заболевания) и «получивших позднее лечение» (получивших медицинскую помощь по истечении 3 месяцев с момента развития заболевания, а также не получивших медицинскую помощь). Значение результирующей переменной – своевременного лечения – получено в ходе регрессионного анализа объясняющих переменных, включающих культурные и эпидемиологические факторы, такие как физические страдания, воспринимаемая причина язвы Бурули, получение помощи извне, а также показания к оказанию медицинской помощи. Среднее время между проявлением симптомов до начала лечения рассчитано в днях. Качественный феноменологический анализ рассказов респондентов прояснил содержательный контекст и динамические характеристики отношения объясняющих переменных со своевременным медицинским лечением.

Результаты

Среднее время начала лечения составило 25 дней для предъязвенных состояний и 204 дня для язв. Потеря дохода и обращение к знахарям продемонстрировали резко отрицательную связь со своевременным лечением. Зачастую респонденты мотивировали обращение к знахарям желанием быстрее выздороветь, чтобы продолжить работать, а также тем, что знахари являлись их родственниками и их помощь была легкодоступна. Однако использование грязной воды для питья в значительной мере связано со своевременным началом лечения, а доступность медицинской помощи способствует своевременному началу лечения (OR 8,5, p=0,012). Полученные данные демонстрируют, что такие факторы системы здравоохранения как доступность медицинской помощи отвечают за соблюдение больными предписанного режима лечения.

Выводы

Полученные данные подчеркивают важность использования комплексного подхода к контролю над распространением ЯБ, учитывающего социальные и экономические особенности, влияющие на несвоевременное лечение, а также факторы, стимулирующие своевременное медицинское лечение. Такой подход должен строиться на периодическом скрининге для выявления случаев заболевания на ранних стадиях, сотрудничестве с частнопрактикующими врачами и целителями, использовании мобильных сервисов для улучшения доступности медицинской помощи, усиления дисциплины в ходе курса лечения и улучшения его результатов.

Translated from English version into Russian by Anna Martynova, through

TRANSLATORS
WITHOUT BORDERS

Factores socio-culturales determinantes de un tratamiento oportuno o tardío de las úlceras de Buruli: implicaciones para el control de enfermedades

Mercy M Ackumey, Margaret Gyapong, Matilda Pappoe, Cynthia Kwakye-Maclean y Mitchell G Weiss

Resumen

Introducción: Los programas de salud pública recomiendan un tratamiento oportuno de las infecciones de úlcera de Buruli (BU) a fin de evitar que las afecciones en estado preulcerativo evolucionen a úlceras y para reducir el número de intervenciones quirúrgicas, discapacidades y el impacto socioeconómico de las BU. Determinar claramente el papel de los factores socioculturales determinantes de un tratamiento médico oportuno puede ayudar a los programas de salud pública a mejorar los resultados de los tratamientos. Este estudio aclaró el papel de los factores socioculturales determinantes y los factores del sistema de salud que inciden sobre las características de un tratamiento oportuno de las BU en una región endémica de Ghana.

Métodos: Se entrevistaron 178 personas con BU mediante una entrevista semiestructurada explicativa basada en el catálogo de modelos de entrevistas explicativas (EMIC). Según la evidencia recogida en las investigaciones, los entrevistados se dividieron en dos grupos: tratamiento oportuno (aplicación de tratamiento médico dentro de los 3 meses posteriores al descubrimiento de la enfermedad) y tratamiento tardío (aplicación de tratamiento médico luego de transcurridos 3 meses de aparición de la enfermedad, o ausencia total de tratamiento médico). La variable de resultado "tratamiento oportuno" se analizó respecto a variables epidemiológicas culturales en modelos de regresión logística para las siguientes categorías: dolor, causas detectadas de las BU, asistencia externa y motivos para el tratamiento médico. La mediana del tiempo transcurrido desde la aparición de los síntomas hasta el inicio del tratamiento se midió en días. Un análisis cualitativo fenomenológico de los relatos de los entrevistados aclararon el significado, el contexto y las características dinámicas de la relación entre las variables explicativas y el tratamiento médico oportuno.

Resultados: La mediana del tiempo para el inicio del tratamiento fue de 25 días para pacientes con preúlceras y 204 días para pacientes con úlceras. El lucro cesante y la consulta con herbolarios son variables que evidenciaron una relación negativa significativa con el tratamiento oportuno. En general, los entrevistados que acudieron a herbolarios fueron motivados por el deseo de recuperarse rápidamente a fin de poder

seguir trabajando y porque los herbolarios eran parientes a los que tenían fácil acceso. Sin embargo, el consumo de agua no potable está relacionado significativamente con el tratamiento oportuno, ya que una vez contactados los servicios de salud se les recomendaba un tratamiento oportuno (o 8,5; p=0,012). Los resultados indican que las variables relativas al acceso a los sistemas de salud determinan el incumplimiento de los planes de tratamiento.

Conclusiones: Los resultados destacan la importancia de un encare global para el control y gestión de las BU que tenga en cuenta las características sociales y económicas que contribuyen a un tratamiento tardío y las que contribuyen a obtener un tratamiento médico oportuno. Este encare debería considerar la implementación de revisiones periódicas que fomenten la detección temprana de casos, la colaboración con profesionales privados y curanderos tradicionales, el uso de servicios móviles para mejorar el acceso, y el cumplimiento de los tratamientos y sus resultados.

Translated from English version into Spanish by Rossana Triaca, through

TRANSLATORS
WITHOUT BORDERS
